# Cancer-Associated Venous Thromboembolism Among Hospitalized Patients with Solid and Hematological Malignancies: A Comprehensive National Study

**DOI:** 10.3390/cancers17050729

**Published:** 2025-02-21

**Authors:** Zaid Khamis, Ghada Araji, Ibrahim Al Saidi, Marian Araji, Chapman Wei, Ahmad Mustafa, Salim Barakat, Varun Chowdhry, Marcel Odaimi

**Affiliations:** 1 Department of Internal Medicine, Northwell Health/Staten Island University Hospital, Staten Island, NY 10305, USA; zkhamis@northwell.edu (Z.K.); ialsaidi@northwell.edu (I.A.S.); sbarakat2@northwell.edu (S.B.);; 2 School of Medicine, Poznan University of Medical Sciences, 61701 Poznan, Poland; marian.araji12@gmail.com; 3 Department of Cardiology, Northwell Health/Staten Island University Hospital, Staten Island, NY 10305, USA; amustafa3@northwell.edu; 4 Department of Hematology/Oncology, Northwell Health/Staten Island University Hospital, Staten Island, NY 10305, USA; modaimi@northwell.edu

**Keywords:** venous thromboembolism, solid malignancies, hematological malignancies, cancer-associated thrombosis

## Abstract

Introduction: Venous thromboembolism (VTE) is a major cause of non-cancer-related mortality in cancer patients. Understanding how demographic factors and cancer types influence VTE risk is critical for developing prevention strategies. This study investigates the incidence of VTE in a large cancer patient population, focusing on gender, race, and differences between solid and hematological malignancies. Methods: Data from the National Inpatient Sample (NIS) database were used to identify cancer patients diagnosed with acute VTE. The patients were divided into those with solid and hematological cancers. Key demographic and clinical characteristics were collected, and patients were matched 1:1 using propensity scoring. Statistical analyses, including logistic regression, assessed VTE incidence and its associations with demographic and cancer type variables. A *p*-value of <0.05 indicated statistical significance. Results: Out of 1,233,832 cancer patients, 63,505 (5.1%) were diagnosed with acute VTE. Females had a higher VTE rate than males (5.5% vs. 4.8%, *p* < 0.001). Racial disparities showed Black patients with the highest incidence (6.4%), followed by White patients (5%). Patients with solid malignancies exhibited a significantly higher incidence of VTE compared to those with hematological malignancies (5.4% vs. 4.1%; *p* < 0.001), with lung cancer and non-Hodgkin lymphoma mostly associated with VTE. Conclusions: This study identifies demographic and cancer-specific differences in VTE risk, emphasizing the need for personalized prevention. High-risk groups, including those with solid tumors, females, and Black patients, may benefit from targeted strategies to reduce the burden of VTE and improve cancer outcomes.

## Simple Summary

Patients with cancer face a high risk of developing blood clots, a condition known as venous thromboembolism (VTE), which can lead to serious health complications or even death. This study analyzed a large group of patients with cancer to understand how factors like cancer type, gender, and race influence VTE risk. The findings highlight significant differences, such as higher VTE rates in females and Black patients. Solid tumors, especially lung cancer, were linked to the highest VTE risk. These insights may help physicians identify high-risk groups and develop tailored strategies to prevent blood clots, improving outcomes for cancer patients.

## 1. Introduction

Venous thromboembolism (VTE) in cancer patients is associated with increased morbidity, mortality, and healthcare costs. It often leads to treatment interruptions, emergency room visits, prolonged hospitalizations, and decreased quality of life [1,2,3]. Recent studies have shown that the risk of VTE in cancer patients is nine-fold higher than the general population [4]. Manifestations of cancer-associated venous thromboembolism include deep vein thrombosis (DVT), pulmonary embolism, and visceral or splanchnic vein thrombosis, together described as VTE [5]. The pathophysiology of cancer-associated venous thromboembolism involves a complex interplay of factors including hypercoagulability, endothelial injury, and stasis, often exacerbated by cancer therapies. The key risk factors can be categorized into patient-related, cancer-related, and treatment-related factors [2,4,6]. Moreover, cancer patients often have multiple risk factors that contribute to the development of venous thrombosis. These include surgical procedures, prolonged hospital stays, immobilization, the use of indwelling catheters, and cancer treatments such as chemotherapy and emerging targeted therapies [7]. Additionally, other comorbidities commonly found in cancer patients will also influence the overall thrombotic risk, as they do in non-cancer patients [8].

Despite advancements in cancer care and the availability of thromboprophylaxis strategies, the landscape of cancer-associated VTE remains complex and ever-evolving. The introduction of novel therapies, including immune checkpoint inhibitors and targeted therapies, has further shifted the epidemiology of VTE in cancer patients. However, there remains a paucity of large-scale studies that comprehensively evaluate the interplay between cancer subtype, demographic factors, and the risk of VTE. In this study, we conducted a detailed analysis of VTE incidence in a large cohort of hospitalized cancer patients. By focusing on differences related to cancer type, site, gender, and race, this study aims to provide insights that can guide risk stratification and the optimization of thromboprophylaxis in cancer patients.

## 2. Methods

We utilized the National Inpatient Sample (NIS) database to identify patients admitted with active cancer from 2016 to 2018. The NIS database is the largest inpatient database in the United States, funded through the Healthcare Cost and Utilization Project (HCUP). IRB is exempt as per the HCUP data use agreement, since patient information is deidentified.

Baseline demographics and comorbidities, including cancer type, were collected using International Classification of Disease 10 codes. Exclusion criteria included patients younger than 18, those with missing data, a prior history of thromboembolism, a history of hypercoagulable diseases, cancer of unknown origin, and those with concomitant solid and hematological malignancies.

The cancer population was stratified into groups based on cancer subtype, gender, and race. The groups were compared to assess the difference in incidence of VTE. Continuous variables were analyzed using student *t*-tests and ANOVAs. Categorical variables were analyzed using chi-square analyses, and Fisher’s exact tests.

The two cohorts of patients with solid malignancies and those with hematological malignancies were then matched in a 1:1 ratio based on age, gender, race, use of antiplatelets and anticoagulants, and active treatment with chemotherapy, immunotherapy, and radiation. Greedy propensity matching was performed using R version 4.1.2 (R Foundation for Statistical Computing, Vienna, Austria). Post-match *p* values were >0.05, signifying significant matching. Binary logistic regression was then performed on the matched cohorts to assess difference in VTE incidence.

All statistical analyses, besides propensity score matching, were performed using IBM SPSS statistics for Windows, version 28 (IBM Corp., Armonk, NY, USA). *p* values < 0.05 were considered statistically significant.

## 3. Results

Out of a total of 1,695,299 cancer patients identified, 1,233,832 were included after exclusions for age under 18, missing data, prior thromboembolism, hypercoagulable disorders, cancer of unknown origin, or co-existing solid and hematological malignancies. The final cohort included 263,427 patients with hematological malignancies (21.4%) and 970,405 patients with solid malignancies (78.6%).

Acute VTE was observed in 63,505 patients (5.1%) across the study population. The incidence was significantly higher in patients with solid malignancies (5.4%) compared to those with hematological malignancies (4.1%) (*p* < 0.001) (Table 1). Of the total VTE cases, 17.2% occurred in patients with hematological malignancies, while 82.8% were in those with solid malignancies (Table 2).

### 3.1. Subtype-Specific Analysis

In solid malignancies, lung cancer had the highest VTE incidence (6.5%), followed by central nervous system (CNS) malignancies (6.1%), and gastrointestinal (GI) cancers (5.4%) (*p* < 0.001). Lung and GI cancers were the most common tumor types accounting for 25.9% and 28.7% of VTE cases, respectively (Table 3).

Among patients with hematological malignancies, VTE incidence varied significantly by subtype. Non-Hodgkin lymphoma had the highest incidence (4.7%), contributing to 42% of VTE cases in this group, followed by Hodgkin lymphoma (4.2%), multiple myeloma (4.0%), myeloid leukemia (3.9%), and lymphoid leukemia (3.4%) (*p* < 0.001) (Table 4).

### 3.2. Matched Analysis

Post-matching analysis, conducted to adjust for confounding variables including age, gender, race, antiplatelet and anticoagulant use, and active treatment, included 522,894 patients (261,447 with hematological malignancies and 261,447 with solid malignancies). VTE incidence remained significantly higher in patients with solid malignancies (5.4%) compared to those with hematological malignancies (4.1%) (*p* < 0.001) (Table 5). Binary logistic regression confirmed this association, with an odds ratio of 1.34 (95% CI: 1.308–1.377, *p* < 0.001).

### 3.3. Gender and Racial Disparities in VTE Incidence

The incidence of VTE was slightly higher among female cancer patients (5.5%) compared to males (4.8%) (*p* < 0.001) (Table 6). Significant racial disparities were observed, with Black patients demonstrating the highest VTE incidence (6.5%), followed by White patients (5.0%), Native Americans and Hispanics (4.9%), and Asians (3.9%) (*p* < 0.001) (Table 7).

A sub-analysis of patients with solid and hematological malignancies was conducted to assess gender and racial differences in VTE incidence. Among patients with hematological malignancies, VTE rates were identical between males and females (4.1%), with no significant difference observed. However, racial disparities persisted, with Black patients exhibiting the highest VTE incidence (4.9%) (*p* < 0.001) (Table 8 and Table 9). In contrast, among patients with solid malignancies, females had a significantly higher VTE incidence compared to males (5.9% vs. 5.1%, *p* < 0.001). Racial disparities remained evident, with Black patients experiencing the highest VTE rates (7.1%, *p* < 0.001) (Table 10 and Table 11).

## 4. Discussion

Several studies have analyzed VTE epidemiology in cancer patients; however, fewer studies have illustrated the difference in the incidence based on individual risk factors. This comprehensive national study provides valuable insights into the incidence of venous thromboembolism (VTE) among cancer patients, highlighting significant variations based on cancer type, site, gender, and race. Our study analyzed a large cohort of cancer patients and observed an overall VTE incidence of 5.1%. This rate aligns with previously reported findings, which indicate a variable incidence ranging from 1.6 to 6.6% [9,10,11,12,13].

Our findings revealed a significantly higher incidence of VTE in patients with solid malignancies compared to those with hematological malignancies (5.4% vs. 4.1%; *p* < 0.001). This association persisted even after matching the cohorts for age, gender, race, use of antiplatelets and anticoagulants, and active treatment with chemotherapy, immunotherapy, and radiation. While limited studies have directly compared VTE incidence between hematological and solid malignancies as broad categories, VTE has historically been more extensively studied and reported in patients with solid tumors. Most prior research focused on comparing specific cancer subtypes rather than general classifications of hematological versus solid malignancies. Even in these studies, hematological malignancies demonstrated a lower VTE incidence compared to several solid tumors [10,14]. Moreover, it have been reported that patients with hematological malignancies have better outcomes after developing VTE compared to those with solid tumors, particularly in terms of lower rates of recurrence, bleeding complications, and mortality [15].

The prothrombotic state inherent to malignancy arises from the interaction of factors associated with Virchow’s triad: stasis, due to prolonged immobility or tumor-induced vascular compression; endothelial injury, caused by cancer cell intravasation, intravascular devices, or systemic treatments; and hypercoagulability, driven by the complex interplay between clinical risk factors, tumor biology, and the host’s physiological response [2,16]. Cancer-mediated hypercoagulability arises from both direct and indirect mechanisms. Cancer cells activate procoagulant pathways through the abnormal expression of tissue factor (TF), the release of TF-expressing microparticles, cancer procoagulants, and other surface particles. Moreover, plasminogen activation inhibitor-1 (PAI-1), a key inhibitor of fibrinolysis, is highly expressed in cancer cells. Indirectly, cancer cells exert systemic effects through the release of proinflammatory cytokines influencing various cell types, including leukocytes, endothelial cells, and platelets, further contributing to hypercoagulability [6,17]. While these mechanisms explain why cancer patients are at an inherently higher risk of VTE, the difference in risk between solid and hematological malignancies remains insufficiently explored. Variations in tumor biology, clinical features, and treatment modalities likely contribute to this disparity. Solid tumors, particularly in advanced stages, often have a higher tumor burden and produce elevated levels of tissue factor in hypoxic microenvironments, which activate the coagulation cascade. Their invasive nature can lead to vascular compression or endothelial damage, directly increasing thrombosis risk. Additionally, treatment-related factors, such as extensive surgeries, thrombogenic chemotherapy agents, and radiation therapy, further exacerbate VTE risk in these patients. In contrast, hematological malignancies, while associated with hypercoagulability, generally lack the direct vessel invasion and mechanical effects seen in solid tumors. Furthermore, conditions like reduced platelet functionality or bone marrow failure in some hematological cancers may counterbalance the risk of thrombosis.

When solid tumors were stratified by site, the highest incidence of VTE was observed in patients with lung, CNS, and GI malignancies. This finding aligns with the results reported in prior studies. While there are some variations in the reported incidence across studies, these types of solid tumors consistently show a significantly elevated risk of cancer-associated thrombosis compared to other malignancies [9,10,13,18,19,20]. This suggests that cancer-specific mechanisms, such as tumor biology and microenvironmental factors, play a central role in predisposing these patients to thrombotic events. Among hematological malignancies, the highest rates of VTE were found in patients with lymphoma, followed by those with multiple myeloma and leukemia. These findings are consistent with previous studies that have documented similar trends in VTE risk within hematological cancers [20,21]. Furthermore, within the lymphoma subgroup, patients with non-Hodgkin lymphoma (NHL) demonstrated higher rates of VTE compared to those with Hodgkin lymphoma (HL). This pattern has also been reported in earlier studies [11,21,22].

Our study demonstrates a significantly higher incidence of VTE in female cancer patients compared to males, specifically in patients with solid malignancies. The role of gender in cancer-associated thrombosis has been inconsistently addressed in the literature. Retrospective studies have reported mixed findings: some have shown that females are at higher risk [13,23], while others, such as Mahajan et al., reported a lower risk of VTE in women across most cancer types [9]. Additionally, Chew et al. found that gender did not significantly predict VTE risk in cancer patients [11]. The observed gender disparity in our study may be attributed to various factors, including hormonal influences, pregnancy-related risks, and differences in cancer types prevalent in each gender. Further research is needed to elucidate the underlying mechanisms and develop gender-specific risk assessment tools.

Our study highlights significant racial disparities in venous thromboembolism (VTE) incidence, with Black patients exhibiting the highest rates. These findings align with prior research reporting increased VTE risk in Black/African American populations compared to other racial groups [9,10,24,25]. While socioeconomic factors and healthcare access have been proposed as contributors, Martens et al. found no direct association between these factors and cancer-associated thrombosis (CAT) risk [10]. Alternatively, disparities in cancer types and stages at diagnosis may play a role. For example, African Americans are more likely to present with advanced-stage lung, breast, and colorectal cancers, as shown in statistical analyses by Krok-Schoen et al. [26]. Additionally, genetic polymorphisms may contribute to the heightened CAT risk observed in Black patients [10]. These findings underscore the importance of further research to elucidate the mechanisms driving racial disparities in VTE and to develop targeted interventions to reduce this inequity.

This study has several limitations that should be acknowledged. First, being retrospective, our findings are subject to potential changes as more data accumulate over time. Second, reliance on ICD 10 codes in the NIS database for selecting patients and classifying comorbidities increases the risk of coding errors. Third, the NIS database includes only inpatient data, which may have introduced selection bias, as certain patient groups might have been more likely to require hospitalization. Additionally, the lack of detailed clinical data, including stage of cancer, treatment, duration of treatment, patient condition when hospitalized, and duration of hospitalization, limits the depth of our analysis. The confounding variables that were not accounted for in our analysis could have influenced the outcomes. Lastly, due to the observational design, causal relationships cannot be firmly established. Future prospective studies and randomized controlled trials are warranted to further explore our findings and their clinical implications.

## 5. Conclusions

Our study, encompassing a large and diverse cohort of U.S. cancer patients, provides a comprehensive overview of cancer-associated VTE incidence, and highlights significant disparities influenced by cancer type, gender, and race. Despite limitations such as its retrospective design and lack of detailed cancer staging or treatment data, our findings emphasize the need for personalized risk assessment models to guide thromboprophylaxis decisions. High-risk groups, including patients with solid tumors, females, and Black patients, may benefit from targeted strategies to reduce VTE burden and improve outcomes. The observed variations underscore the inadequacies of current thromboprophylaxis guidelines and support the development of nuanced, cancer-specific recommendations tailored to malignancy type and patient demographics. Future prospective studies are essential to validate these findings, elucidate underlying mechanisms, and develop effective, targeted interventions for optimizing VTE prevention and management in cancer patients.

## Figures and Tables

**Table 1:** Cancer subtype distribution and VTE incidence.

Cancer Type	Number of Patients	Percentage of Total Patients (%)	Patients with VTE	VTE Incidence (%)	*p*-Value
Hematological malignancies	263,427	21.4%	10,901	4.1%	*p* < 0.001
Solid malignancies	970,405	78.6%	52,604	5.4%	
Total	1,233,832	100%	63,505	5.1%	

**Table 2:** Distribution of VTE cases by cancer subtype.

Cancer Type	Number of VTE Cases	Percentage of Total VTE Cases (%)
Hematological malignancies	10,901	17.2%
Solid malignancies	52,604	82.8%
Total	63,505	100%

**Table 3:** VTE incidence in solid malignancy subtypes.

Solid Malignancy	Number of Patients	Percentage of Total Patients	Patients with VTE	VTE Incidence	Percentage Within Acute VTE	*p*-Value
Lung	209,376	21.6%	13,611	6.5%	25.9%	*p* < 0.001
Central Nervous System	30,490	3.1%	1851	6.07%	3.5%	
Gastrointestinal	277,724	28.6%	15,114	5.44%	28.7%	
Genitourinary	93,288	9.6%	4849	5.19%	9.2%	
Skin and Soft Tissue	42,589	4.4%	2117	4.97%	4%	
Gynecological	179,909	18.5%	8672	4.82%	16.5%	
Breast	90,761	9.4%	4346	4.78%	8.3%	
Head and Neck	37,271	3.8%	1666	4.46%	3.2%	
Bone	8999	0.9%	378	4.20%	0.7%	
Total	970,405	100%	52,604	5.4%	100%	

**Table 4:** VTE incidence in hematological malignancies subtypes.

Hem Malignancy	Number of Patients	Percentage of Total Patients	Patients with VTE	VTE Incidence	Percentage Within Acute VTE	*p*-Value
Non-Hodgkin Lymphoma	96,528	36.6%	4579	4.7%	42%	*p* < 0.001
Hodgkin Lymphoma	8522	3.2%	357	4.2%	3.3%	
Multiple Myeloma	56,160	21.3%	2230	4.0%	20.5%	
Myeloid Leukemia	46,246	17.6%	1813	3.9%	16.6%	
Lymphoid Leukemia	55,971	21.2%	1922	3.4%	17.6%	
Total	263,427	100%	10,901	4.1%	100%	

**Table 5:** Post-match analysis results.

	VTE Cases	VTE Incidence	Total	*p*-Value
Hem malignancies	10,761	4.1%	261,447	*p* < 0.001
Solid malignancies	14,238	5.4%	261,447	
Total	24,999	4.8%	522,894	

**Table 6:** Gender distribution and VTE incidence.

Gender	Number of Patients	Percentage of Total Patients (%)	Patients with VTE	VTE Incidence (%)	*p*-Value
Males	662,188	53.7%	31,824	4.8%	*p* < 0.001
Females	571,644	46.3%	31,681	5.5%	
Total	1,233,832	100%	63,505	5.1%	

**Table 7:** Race distribution and VTE incidence.

Race	Number of Patients	Percentage of Total Patients	Patients with VTE	VTE Incidence	*p*-Value
White	881,320	71.4%	43,731	5%	*p* < 0.001
Black	159,690	12.9%	10,395	6.5%	
Hispanic	107,360	8.7%	5260	4.9%	
Asian or Pacific	39,727	3.2%	1543	3.9%	
Native American	5435	0.44%	267	4.9%	
Others	40,300	3.26%	2309	5.7%	
Total	1,233,832	100%	63,505	5.1%	

**Table 8:** Gender distribution and VTE incidence in hematological malignancies.

Gender	Number of Patients	Percentage of Total Patients (%)	Patients with VTE	VTE Incidence (%)	*p*-Value
Males	147,387	56.3%	6058	4.1%	*p* < 0.868
Females	114,060	43.6%	4703	4.1%	
Total	261,447	100%	10,761	4.1%	

**Table 9:** Race distribution and VTE incidence in hematological malignancies.

Race	Number of Patients	Percentage of Total Patients	Patients with VTE	VTE Incidence	*p*-Value
White	188,064	71.9%	7592	4%	*p* < 0.001
Black	31,038	11.9%	1507	4.9%	
Hispanic	24,225	9.26%	967	4%	
Asian or Pacific	7347	2.81%	210	2.9%	
Native American	910	0.34%	38	4.2%	
Others	9863	3.77%	447	4.5%	
Total	261,447	100%	10,761	4.1%	

**Table 10:** Gender distribution and VTE incidence in solid malignancies.

Gender	Number of Patients	Percentage of Total Patients (%)	Patients with VTE	VTE Incidence (%)	*p*-Value
Males	147,387	56.3%	7462	5.1%	*p* < 0.001
Females	114,060	43.6%	6776	5.9%	
Total	261,447	100%	14,238	5.4%	

**Table 11:** Race distribution and VTE incidence in solid malignancies.

Race	Number of Patients	Percentage of Total Patients	Patients with VTE	VTE Incidence	*p*-Value
White	188,064	71.9%	9817	5.2%	*p* < 0.001
Black	31,038	11.9%	2203	7.1%	
Hispanic	24,225	9.26%	1244	5.1%	
Asian or Pacific	7347	2.81%	320	3.4%	
Native American	910	0.34%	31	4.2%	
Others	9863	3.77%	623	6.3%	
Total	261,447	100%	14,238	5.4%	

## Data Availability

While the datasets in the Healthcare Cost and Utilization Project are public and available through the HCUP Central Distributor, data availability is restricted to people approved by the Agency for Healthcare Research and Quality to purchase the data. To receive approval from the Agency of Healthcare Research and Quality to purchase the data, applicants must complete the HCUP Data Use Agreement Training and sign an HCUP DUA. The datasets can be requested when accessing the online HCUP Central Distributor. Information regarding purchasing this publicly available database can be found at https://hcup-us.ahrq.gov/tech_assist/centdist.jsp. Accessed on 1 November 2024.
